# Technologies of Production of Materials Based on WPC: A Short Review

**DOI:** 10.3390/polym17081025

**Published:** 2025-04-10

**Authors:** Zuzana Mitaľová, Juliána Litecká, Marek Kočiško, Khrystyna Berladir

**Affiliations:** 1 Department of Automobile and Manufacturing Technologies, Faculty of Manufacturing Technologies, Technical University of Kosice, Bayerova 1, 08001 Presov, Slovakia; khrystyna.berladir@tuke.sk; 2 Department of Physics, Mathematics and Techniques, Faculty of Humanities and Natural Sciences, University of Presov, 17 November Street—No. 1, 08116 Presov, Slovakia; juliana.litecka@unipo.sk; 3 Department of Computer Aided Manufacturing Technologies, Faculty of Manufacturing Technologies, Technical University of Kosice, Bayerova 1, 08001 Presov, Slovakia; 4 Department of Applied Materials Science and Technology of Constructional Materials, Faculty of Technical Systems and Energy Efficient Technologies, Sumy State University, Kharkivska 116, 40007 Sumy, Ukraine

**Keywords:** wood plastic composite, production technology, wood flour, polymer, biodegradable thermoplastic

## Abstract

This paper review deals with frequent technologies of the production of materials based on Wood Plastic Composite, their brief definition, and description of components. The choice of processing technology depends on the polymer applied and the shape required of the part or the component. In the case of thermoplastic matrices, the dominant are extrusion and injection molding. In the case of thermosets application, the following technologies can be used: Resin Transfer Molding and Sheet Molding Compound. Currently, the research is also widely focused on composites with a matrix made of biodegradable thermoplastics—polylactide, which also brings to the forefront 3D printing technology of Fused Deposition Modeling. Each of these technologies is—to a certain extent—limited and impacts on the final characteristics of the composite material and its use.

## 1. Introduction

Wood Plastic Composite (WPC) lacks a universal definition; it is a kind of an engineering composite material produced by mixing wood fibers or flour (other lignocellulose fibers) with thermosets and thermoplastics. WPC-based composite materials represent an advanced alternative to natural materials/plastics and combining the advantages of both components. Following this combination of advantages, their application is possible: decorative and acoustic elements in construction, decking, fencing, for production of interior/exterior furniture and flooring (due to resistance to moisture, steam, deformation, and minimal maintenance requirements), automobile components, consumer goods, etc. In relation to a wide field of application, it is necessary to pay attention to the selection of a suitable technological method.

## 2. Components of WPC-Based Materials

**Figure 1:**
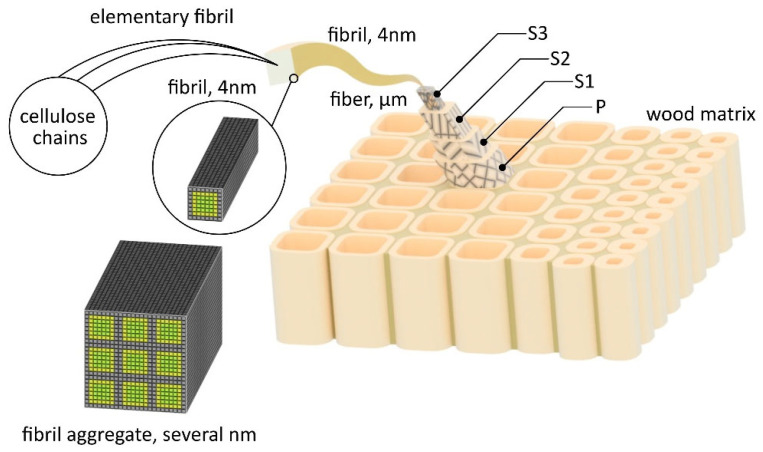
Structure of wood fiber, various layers (S1, S2, S3) in wood cell wall.

Wood fiber (WF) is generally made from timber by chemical, mechanical, biological, and many combined processes. Wood is a fibrous, organic, heterogeneous, anisotropic, hygroscopic, biodegradable substance with a cellular structure, sufficient strength, good machinability and affordability. From the chemical point of view, wood consists of approx. 40–50% cellulose, 15–25% hemicellulose, 15–30% lignin + resins, tannins, and other solids in small quantities. In general, WFs are applied as reinforcement of plastic matrices due to their high specific strength, thermal insulation, and low density. Hydrophilicity (ability to absorb H_2_O), low UV resistance, high flammability index, and insufficient interphase adhesion of composite material (CM) components can be modified by physical and chemical procedures. WFs consist of four layers: middle lamella: M, primary wall: P, secondary wall: S (including: outer layer—S_1_/the middle layer—S_2_/inner layer—S_3_), and warty layer: W, see Figure 1. The individual layers and their composition are described in detail in the study [1]. Similarly, wood flour—derived from various wood planer shavings, chips, sawdust, and other residues of the woodworking industry—can be applied as matrix reinforcement. In fact, wood flour is much more widely available than fibers. On the other hand, a study [2] shows a direct correlation between the wood species (hardwood/softwood) and the resulting mechanical properties of WPC-based materials. Due to the higher cellulose content, samples made from hardwood exhibit higher values of tensile elongation at break and Charpy impact strength [1,2,3,4].

In the process of manufacturing WPC-based composite materials, it is necessary to ensure a processing temperature of approximately 200 °C (some studies indicate a temperature up to 220 °C). This is due to the susceptibility of wood fibers to thermal decomposition, which leads to deterioration of mechanical and organoleptic properties. In relation to this requirement, thermoplastic matrices such as PP, PE, PVC, or PS have been used for a long time now. The use of WPC with a thermoplastic matrix is wide-ranging—from the automotive industry (for example: door panels, gearshift knobs), to construction (decking), siding, window frames, fencing products, furniture, and technical applications. The viscosity of thermoplastic matrices is about 500–1000 times higher compared to the viscosity of uncured thermoset resins. These matrices also have higher impact resistance, higher damage tolerance, and are chemically inert. Many thermoplastic matrices can be used with a full range of properties. Thermosets—polyesters, epoxies, and vinyl esters—are also being applied. These types of resins have higher modulus, improved creep resistance, higher thermal and chemical stability (than thermoplastics), but are fragile at room temperature [3,5,6,7,8].

Studies are currently underway to verify the use of PMMA, Nylon 6, and bio-degradable polylactic acid (PLA) or polyhydroxyalkanoate (PHB)-based polymers. The reason for the application of bio-based polymers is to reduce dependence on petroleum products also in this segment of composite materials production. Other interesting matrices can be obtained from starch and cellulose [3,5,6,7,8].

Wood fibers (flour) and plastic matrix represent the main components of wood–plastic. Both components can be used in their primary and recycled form—recycled PVC bottles, organic cellulose waste, and wood processing industry. The share of reinforcement in commercial WPC products varies. In addition to the above-mentioned components, various types of additives are applied to modify mechanical properties, simplify the production process, and increase rheological properties, from functional—e.g., compatibilizers that ensure “binding” at the components’ interface—to pigments that create the desired shade and ensure the aesthetic side of the product. Mechanical properties of WPC composites depend on several aspects [6,9,10]:

- the type of polymer matrix applied;
- the percentage share of organic reinforcement—in addition to wood (it is possible to use plant fibers);
- morphology, physical properties, moisture content of the reinforcement particles themselves;
- quantity of individual additives;
- origin of input raw materials (location of natural fiber collection, type of tree, possibility of applying recycled material, etc.);
- technology and production conditions.

Significant milestones in the history of WPC [9,10,11,12]:

- 1906—first mention of the application of wood flour in a composite called Bakelite;
- 1983—production of materials for car interiors by Lear Corporation (Sheboygan, Wisconsin), a blend consisting of 50% PP + wood flour;
- early 1990s—Advanced Environmental Recycling Technologies (Texas) in cooperation with the division of Mobil Chemical Company (Virginia) produced WPC material with a high content of wood fibers;
- 1991—first conference on organic-filled plastics was held in Madison (Wisconsin);
- 1993—Andersen Corporation (Minnesota) started the production of wood-filled plastics with PVC matrix—applied to sub sills for French doors (the components contained 40% wooden reinforcement);
- 1996—the beginning of pellets production to produce WPC (a few U.S. companies).

Lately, a 50% increase in interest in WPC from the field of construction has been noted. Over the same time horizon, there has been a 15% increase in the interest of automotive manufacturers in materials with natural reinforcement. A step towards reducing the carbon footprint from transport is to reduce the weight of the vehicle—just by changing its material composition. The study [13] shows a direct correlation between the weight of the vehicle and fuel savings. A 10% reduction in vehicle weight saves between 6 and 8% of fuel (about 5–10 kg natural reinforcement is used per vehicle). Another reason for their application is the socio-ecological aspect. Materials based on WPC have lower environmental impact compared to CM-reinforced glass fibers. At the same time, farmers are offered the opportunity to grow other than cultivated crops, namely crops for the production of natural reinforcements. Although WPC-based products have now become fully established as quality materials, they continue to remain relatively new products compared to other materials that are being substituted [5,13,14].

## 3. WPC Production Technology

In relation to production, WPC composite materials are flexible and have a good strength-to-weight ratio. The characteristics of the product are (among other things) also crucially influenced by the production technology. Frequent production technologies of WPC composite materials with thermoplastic matrices include the following [6,15,16,17,18,19]:

- extrusion—for linear profiles, suitable technology for polymers with high molecular-weight, dominant technology in the production of WPC products in decking application and building industry;
- injection molding—for three-dimensional parts of regular and irregular shapes;
- compression molding—inexpensive production of complex parts, possibility to add special reinforcements in the production process, no need for skilled personnel;
- it is also possible to apply the little-mentioned rolling method—the so-called calendering—to the production of floorings.

**Figure 2:**
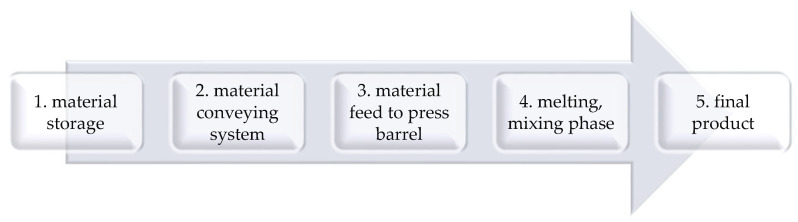
Phases of the production process: 1. material storage in bags, big bags, and silos; 2. pneumatic or mechanical material conveying system; 3. material feed to the press barrel (continuous/discontinuous filling of the press barrel); 4. melting and mixing phase; 5. final product and control.

Before the extrusion/injection molding process itself, it is necessary to pass through the stages of the process—see the diagram (Figure 2).

Where thermosets are applied as WPC matrices, it is possible to use injection molding technology, referred to as RTM—Resin Transfer Molding and SMC—Sheet Molding Compound Technology [15,16,17,18,19].

The key to a successful WPC product production is perfect distribution and dispersion, i.e., dispersion of the wood component in the plastic matrix. In the literature, this process is also referred to as compounding. Hydrophilic wood fibers and hydrophobic polymer require an input of physical energy (via shear forces) to homogeneously mix the two components. Dispersion can be increased by the application of binders or dispersing agents, but it is primarily ensured by mechanical mixing of the components. To create a homogeneous blend, the screw mechanism of the extrusion press is used directly. If the blend needs pre-mixing, mixers are applied. E.g., intensive shear mixers achieve blend formation before injection molding. Insufficient aggregation causes poor dispersion of the plastic and poor wetting of the fibers [17,20,21].

**Figure 3:**
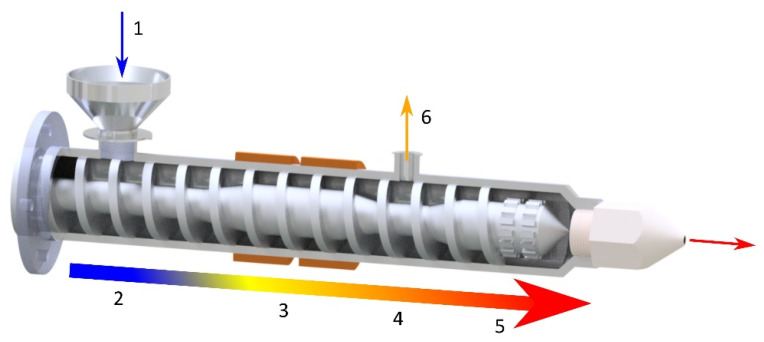
Cross-section of single-screw press and material flow: 1—product feed, 2—feed zone, 3—melting zone, 4—degassing zone, 5—metering zone with mixing head, 6—degassing.

**Figure 4:**
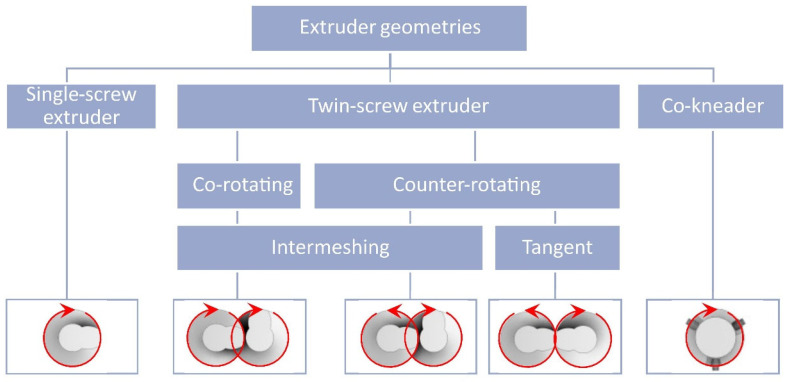
Overview of extruder systems for WPC production.

**Figure 5:**
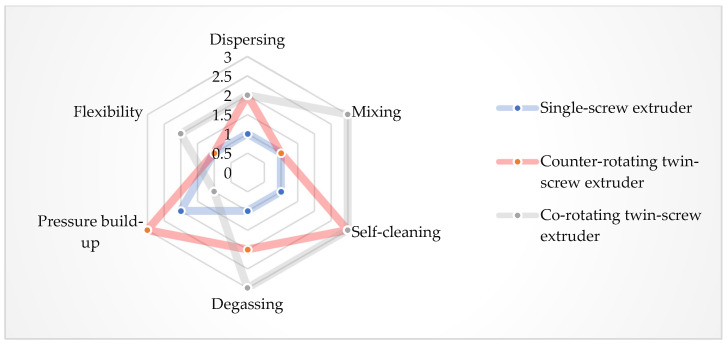
Comparison of different extruders systems (3—very good, 2—good, 1—satisfactory).

The material is fed into the extruding machine in the form of granules, pellets, or powder. A screw or screws convey the material into the extruder, whose task is to melt the polymer, mix the individual components, and allow the blend to be transported to the die of the press (extrusion phase of the final product). Several types of extruders can be used in the WPC production: a single-screw extruder (cross-section of single-screw extruder with individual zones—see Figure 3), twin-screw extruder with co-rotating or counter-rotating screws, co-kneader and Woodtruder^TM^. For the overview and comparison of extruder systems, see Figure 4 and Figure 5. Hollow, solid profiles or sheet materials and other semi-finished products are examples of typical products manufactured with extrusion [15,22].

Table 1 describes the pros and cons of single- and twin-screw extruders. A co-kneader is so-called a self-cleaning extruder. It is a single-screw extruder with pins—fixed on the barrel of the extruder (pins “clean” the surface of the extruder screw). Advantages of this system are a lower energy consumption, lower melt temperatures, maintained homogeneity of the blend, and high production rate [17,23,24].

Woodtruder^TM^ is a system with an acquisition cost, which consists of a twin-screw extruder with counter-rotating screws (with ratio: $\frac{l}{d}=\frac{28}{1}$) + a single-screw extruder, control system, a die tooling system, cooling tank, a saw, and run-off table. The advantages of the Woodtruder^TM^ are as follows [24]:

- no pre-treating of the material by drying required (moisture is removed by a vacuum aeration mechanism) and mixing;
- ability to keep the melt temperature low with high head pressure;
- integrated process control system (feeding + extruder unit operations).

To achieve the required product quality, it is necessary to select optimal parameters of the extrusion process: screw speed *n* (rpm), feed rate *Q* (kg·h^−1^), temperature of individual heating zone and extrusion die, respectively. The type of extruder and the design of the screw also play a role. To produce short-fiber composites, a twin-screw extruder is recommended, whereas a single-screw extruder is preferred for processing long-fiber composites (due to the reduced shear rate). The optimization of process parameters is complex, as it relates to variations in the final composite—possible combinations of different matrix types and fibers. The compatibility and porosity of composite components are not primarily dependent on the set production parameters but rather on the pretreatment of natural fibers [25].

### 3.1. Injection Molding

Injection molding is applied in mass production and to complex shapes of WPC-based products, using predominantly horizontal presses. The equipment for this production technology requires high input costs but allows for a high degree of automation and requires little maintenance. The advantage of the technology is the shortness of the production cycle, compliance with the required dimensional tolerances, and a low number of additional secondary machining operations. Injection molding is suitable for low molecular weight and low viscosity polymers. In this method, the polymer is heated to the forming temperature, and under the influence of pressure, it flows into a mold cavity where its solidification occurs.

The process is cyclical, discontinuous, and takes from a few to several ten seconds depending on the size of the part and the time needed for cooling. The injection cycle consists of a series of specific precision operations (phases); it is a non-isothermal process where the blend undergoes a temperature cycle. Phase 1—mold closure, phase 2—injection of the blend into the mold cavity (during this phase 90–95% of the compound is injected), phase 3—additional pressure—to compensate for shrinkage, phase 4—final cooling and ejection. The difference between molding WPC by injection and injection molding of a pure thermoplastic is that thermoplastic is reversible (it can be converted to a liquid state after cooling by reheating), while the WPC blend is irreversible (there is a permanent change in the volume and properties of the wood filler). Injection molding technology is not suitable for WPC materials with high content of wood fiber [15,16,26,27].

In moldings with long fibers, microcracks at cooling occur because the matrix shrinks more than the filler. A volumetric change in the matrix will cause stress in the material that is greater than the bonding force at the matrix/filler interface. In relation to production technology, wooden particles are placed in the direction of the polymer flow. Studies report that composite materials made by injection molding (PP matrix + natural fibers) show better mechanical properties (tensile strength/flexural strength/impact resistance)—compared to the same type of material made by the pressing technology. As with other production technologies, it is necessary to ensure moisture control. The high moisture content of the filler may cause a build-up of vapor in the press chamber with subsequent vapor explosion, leading to damage or gradual wear of the screw. Injection-molded parts are used in the automotive industry (for fixing hooks, sound systems, and glove boxes), furniture industry, and consumer goods, too [3,9,27,28,29].

Extrusion and injection can run as a one-step (direct) or a two-step process. Direct extrusion/injection combines the component mixing phase and product forming in one step. In the case of a two-step process, a middle product (granulate) is made, and then the desired shape is acquired in the next step (cooling occurs between the first and the second step) [30].

The injection molding system contains three units with different requirements: feed zone (temperature about 30 °C), heating zone (variable temperatures, standard between 125 and 160 °C), and injection nozzle (temperature about 155 °C). The optimum polymer flow rate is 20 cm^3^/s, and the injection pressure is 120 MPa to ensure proper shaping. Temperatures may vary depending on the type of fiber and the matrix of the composite [31,32].

### 3.2. Compression Molding

**Figure 6:**
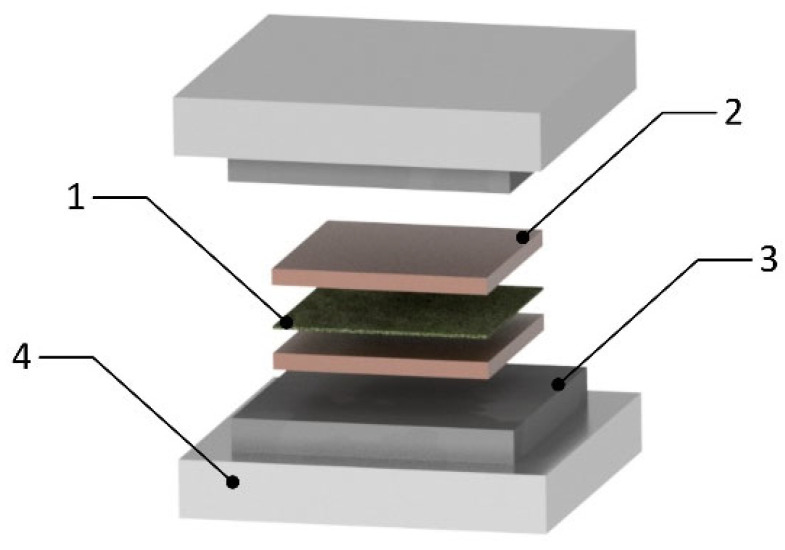
Scheme of compression molding: 1—natural fiber material, 2—thermoplastic sheet, 3—steel mold, 4—heating stage.

Compared to injection molding, compression molding technology is more versatile and low-cost (but not suitable for thin parts). In the case of the production of parts, it is possible to apply all forms of fibers, from short, through long, to fabrics, with the possibility of adding reinforcements during the production process. Natural fiber materials can be sandwiched or laminated between polymer sheets. No skilled personnel are required for production (workers are not exposed to toxic chemicals). A typical compression molding machine consists of two stages: top and bottom, which can be heated and cooled to the desired temperature. A schematic representation of compression pressing is shown in Figure 6. A balance between temperature, pressure, and molding time must be ensured in the process. The process temperature should not exceed the degradation temperature of natural fibers (*T_max_* = 200–220 °C). Several studies indicate that pressure (rather than temperature) has a more significant impact on the final mechanical properties. When the pressure increased, tensile strength values increased. The application of higher temperatures leads to weakening of the samples—likely due to the beginning of decomposition of the natural fibers. Table 2 shows the applied parameters of compression molding technology for composites with a thermoplastic matrix, obtained from various research sources. In the case of varying parameters between pressure and temperature, the optimal parameters for achieving the desired properties, as specified by the author, are provided.

At the same time, it is necessary to maintain the moisture content of the natural fibers at a level of up to 3 wt. %, as the residual moisture in the fibers may cause bubbles and voids in the final composite [17,18].

### 3.3. Fused Deposition Modeling

**Figure 7:**
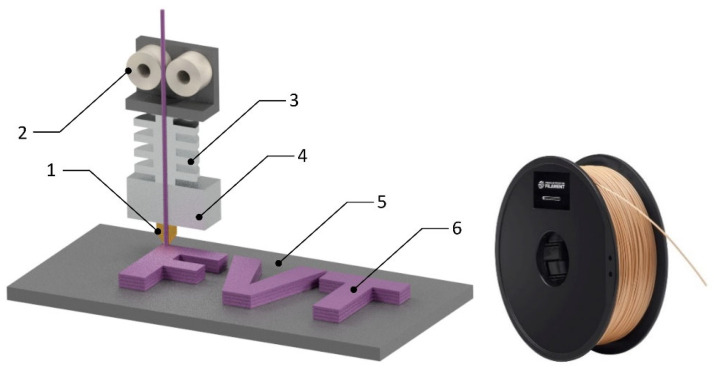
Left side: schematic of FDM printing process: 1—nozzle, 2—feeder wheels, 3—heat sink, 4—hot block, 5—bed, 6—printed part; right side: filament—PLA matrix + wood derivates includes wood dust, cork, bamboo (producer of filament: 3DPBlock) [42].

In the case of the WPC composite thermoplastic matrix, it is also possible to apply 3D printing technology—FDM (Fused Deposition Modeling), operating on the principle of layering the molten filament point by point—up to the final three-dimensional object—see Figure 7. In the production of filaments, wood dust is applied in conjunction with a plastic matrix, the presence and proportion of which affects the quality of the printed part [38]. The study [39] describes the production of tensile test specimens. In the case of the specimens made, errors in the volume (microcracks and macrocracks in the cross-section between the layers) and unwanted concave curvature of the samples were detected. Studies [40,41] point to inhomogeneity and porosity of printed specimens (despite different matrices and reinforcements of the filament).

Currently, the research is also widely focused on composites with a matrix of biodegradable thermoplastics—e.g., polylactide—PLA (these composite materials are often referred to as bio-composites). A lower content of wood particles in the bio-composite leading to a lower stress concentration results, thus resulting in higher tensile strength. Several authors have studied the mechanical properties of PLA/versus wood prints. The application of wood particle filaments (or natural reinforcements in general) in conjunction with PLA matrix in 3D–FDM printing has become increasingly attractive due to their low environmental impact and cost of material [43,44,45,46,47].

### 3.4. Applied Technologies for WPC-Based Materials with Thermosets Matrix

When it comes to applying thermosets (as WPC matrices), it is possible to use injection molding technology, referred to as RTM—Resin Transfer Molding and SMC—Sheet Molding Compound technology. Descriptions of these technologies are shown in Table 3.

### 3.5. WPC Foams Production Technologies

WPC foams production technologies: the three production technologies mentioned (extrusion, injection molding, and compression molding) can be modified to produce WPC foams. From the information above, the WPC production process often includes two steps: compounding and product forming (in some cases these steps run continuously). Foaming agents are added to the process in the second step. Their application reduces the manufacturing cost and the weight of the final product, its shrinkage/deformation (ensuring profile shape accuracy), and improves impact strength and physical properties.

Special processing equipment or method is often required to handle the blowing agents (CBAs—Chemical Blowing Agents, PBAs—Physical Blowing Agents) and make WPC foams [9,17,56,57].

### 3.6. Future Trends

Many manufacturers and sellers claim that composite materials with natural reinforcements are recyclable and refer to them as “eco-friendly materials.” This information is often misinterpreted and can mislead customers. Primarily, the raw materials—natural fibers—are recyclable, and for matrices, recyclability depends on the type of plastic used. A challenge in the production of natural fiber-based composites is the application of biodegradable matrices (e.g., PLA—polylactide acid, PHA—polyhydroxyalkanoate, PHB— polyhydroxybutyrate, etc.) in various technologies, correctly setting process parameters, and addressing emerging issues. A starting point for eliminating “defects” in the final component could be the simulation of individual production processes.

Other challenges in these composites include further improving the compatibility of components during the manufacturing process—such as continuous modification of natural fibers (e.g., alkalization to reduce moisture absorption, etc.) and expanding their application into other industries such as construction, aerospace, marine applications, sports equipment, and more.

## 4. Conclusions

Currently, the automotive industry is experiencing a growing demand for the aforementioned composite materials. The primary driver behind this trend is the need to reduce vehicle fuel consumption and mitigate greenhouse gas emissions by lightweighting structural components. This can be achieved through the application of composite materials reinforced with natural fibers. Such components offer several advantages, including relatively low cost, reduced weight, sufficient stiffness, and non-toxicity. A secondary motivation is the reduction in dependency on petroleum-based products, thereby lowering the carbon footprint. Additionally, the use of locally cultivated plants for fiber production presents an economic opportunity by fostering collaboration between local farmers and composite manufacturers. The selection of appropriate manufacturing technology is influenced by multiple factors—primarily the geometry and shape of the component, production volume, and cost considerations. Key parameters also include the type of applied matrix, processing temperature, and the mechanical properties of the final product. A major challenge in this field is the implementation of biodegradable matrices, which has significant implications for the recyclability of natural fiber-reinforced composites. Currently, the recycling of these materials remains in the early stages of research and development.

## Figures and Tables

**Table 1:** Extruder types—pros and cons [17,23,24].

Single-Screw Extruder (Screw Speed Range: 60–250 rpm)		
Designed by German machine manufacturer Paul Troestar in 1935, primarily for thermoplastics. The design of the extruder is relatively simple, suitable for both extrusion and injection molding. Barrel length to diameter ratio: $\frac{l}{d}=\frac{34}{1}$. The materials are fed into the extruder via a gravity hopper (feeders are not required). To remove unwanted volatile substances, the extruder is equipped with a vent unit.		
**Pros**	**Cons**	**Dynamic Principle**
Low investment costs, proven production technology.	Necessity of a pre-treatment phase: drying, granules preparation—premixed blend—(application of thermokinetic mixers), low production rate, high screw speed, inability to maintain low melt temperature with higher head pressure.	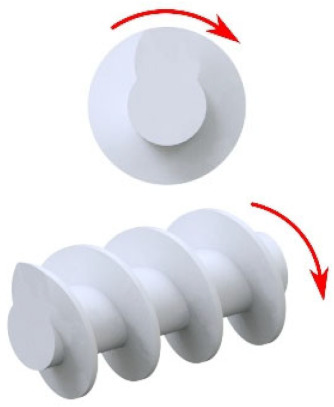
**Twin-Screw Extruder with Co-Rotating Screws (Screw Speed Range: 100–1200 rpm)**		
Werner & Pfleiderer (nowadays Coperion) delivered the first co-rotating, closely intermeshing ZSK twin-screw extruder in 1957. The ZSK designation is derived from the German name Zwein Schnecken Kneter and has long been synonymous with this type of extruder. The materials are fed into the extruder using double-screw side feeders or gravimetric feeders. To remove moisture from the process, the extruder is equipped with atmospheric vacuum vents.		
**Pros**	**Cons**	**Dynamic Principle**
No material pre-treatment required (fibers with a moisture content of 6% ± 1 can be applied), variable drives.	High speed of screw rotation, no screw cooling, inability to maintain low melt temperature with higher head pressure.	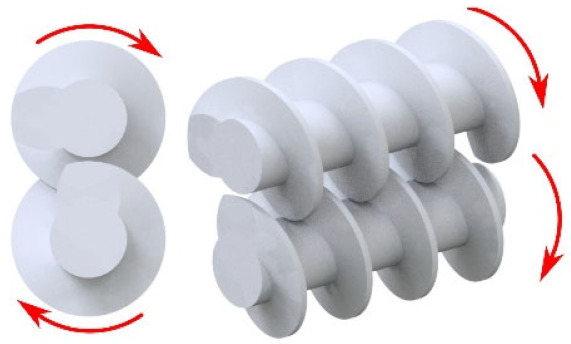
**Twin-Screw Extruder with Counter-Rotating Screws (Screw Speed Range: 25–80 rpm)**		
Especially for WPC products with PVC matrices that need degassing during extrusion process. Their screw configuration is either parallel or conical.		
**Pros**	**Cons**	**Dynamic Principle**
Low screw revolution per minute (rpm) → reduces the risk of burning the materials, low induced shear produced by mixing process, proven production technology.	Requires a blend-drying system, mixing the blend, higher purchase price (compared to a single-screw press), and operating costs.	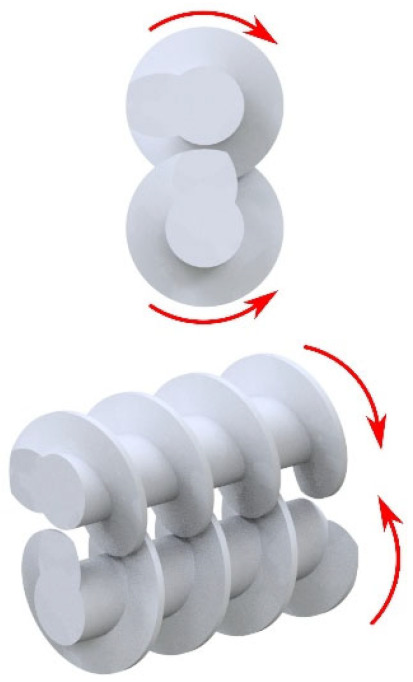

**Table 2:** Process parameters of compression molding technology for CMs with natural reinforcement and PP matrix [33,34,35,36,37].

Type of Fiber/ Volume of Fiber (wt%)	Pressure	Temperature (°C)	Study/References
Hemp/jute/sisal	0.5/1.0/1.5	165/175/185	Yallew, et al. (2020) [33]
The optimal parameters for achieving the desired properties → pressure 1 MPa/temperature 175 °C.			
Bamboo	0 ton (5 min), 4 tons (1 min), 5 tons (1 min)	200	Ovlaque, et al. (2020) [34]
The optimal parameters for achieving the desired properties → not specified.			
Sisal/50, 60, 70, 80	13.8 MPa	–	Prajwal, et al. (2019) [35]
Coir/0, 10, 15, 20	30 KN	170	Mir, et al. (2013) [36]
Hemp	0.8 MPa	160–190	Takemura and Minekage (2012) [37]
The optimal parameters for achieving the desired properties → temperature below 180 °C.			

**Table 3:** Applied technologies (matrix: thermoset) [18,48,49,50,51,52,53,54,55].

RTM Injection	Study/References
Forming of composite parts carried out using a closed mold. The low-pressure pump injects resin and hardener into the mold. Subsequently, the blend is injected into the reinforcement pre-form. The resulting part is cured at room temperature (or above till the end of the curing reaction). The method is applied to the production of car interior parts with relatively high strength, rigidity, and dimensional stability. Wood chips with a high ratio of $\frac{L}{d}=350-400$ are used for reinforcement, similarly to OSB boards. This high aspect ratio causes efficient transfer of shear forces from the matrix to the fiber. The advantage in the application of natural fibers (compared to synthetic ones) is their ability to act as an “absorber” of the resin to → absorb fluids as it flows (consequence—natural fiber preforms require additional injection time). The advantages of the RTM technology are a short production time (compared to hand lay-up technology), low clamping pressure, no need for skilled personnel, excellent surface finish, the possibility of producing complex geometries. Disadvantages: high-cost process of production and limited to manufacturing of small-dimensional parts. A critical aspect of the RTM process is mold filling, especially for parts with greater thickness. Models simulating resin flow in the mold can prevent defects such as voids or poor wetting of the fibers, similarly to the application of resins with low viscosity values (to ensure proper fiber wetting—e.g., epoxy, phenolic, polyester/acrylic). The company Bcomp provides specially spun fibers PowerRibs™/ampliTex™ in prepreg form, applicable for the production of automotive parts (as well) via RTM technology. These are a replacement for monolithic carbon fiber car parts, offering the same stiffness but reduced weight. Additionally, the newly created prepreg (made from natural fibers and resin) is formable and produces no toxic waste.	Mitaľová, et al. (2023) [48] Gartner, et al. (2022) [49] Kim and Pal (2010) [50] Lim and Lee (1999) [51]
**SMC**	Elseifi, et al. (2021) [52] Orgéas and Dumont (2012) [53] Voorn, et al. (2001) [54] Ren, et al. (2009) [55]
Method of pressing a composite blend with short fibers saturated with a thermoset matrix. The process consists of two steps: prepreg production and compression (pressing). Natural-based fibers are good substitution candidates to glass fibers for SMC. Several studies support the claim regarding the mechanical properties of natural-fiber-reinforced SMC material. The study by Voorn, et al.—application of flax fiber SMC materials—as a replacement for glass fibers—achieved approximately 20% lower weight, with relatively the same stiffness, but lower impact property values (caused by the anisotropic nature of natural fibers). Similarly, the study by Ren, et al. showed tensile strength values (44 MPa) and Young’s modulus of elasticity (14 GPa), similar to GFSMC composites. The SMC technology is frequently applied in the automotive industry for the production of bumpers, trunk covers, and spoilers.	
**Pressing**	Hodzic and Shanks (2014) [18]
Processing of the semi-finished product by the application of pressure and temperature: measured amount of pressed mass is fed into the heated mold and then, under the action of the aforementioned factors, it turns into a liquid state, fills the mold, and hardens. Mechanical properties of the WPC molding are influenced by the mold cavity design and the process parameters—blend temperature, mold heating and cooling, mold closing speed, etc. (technology also suitable for thermoplastics).	

## Abbreviations

The following abbreviations are used in this manuscript:

CBAs	Chemical Blowing Agents
CM	Composite Material
FDM	Fused Deposition Modeling
GFSMC	Glass Fiber Sheet Molding Compound
PBAs	Physical Blowing Agents
PE	Polyethylene
PHA	Polyhydroxyalkanoate
PHB	Polyhydroxybutyrate
PLA	Polylactide Acid
PMMA	Polymethylmethacrylate
PP	Polypropylene
PS	Polystyrene
PVC	Polyvinylchloride
RTM	Resin Transfer Molding
SMC	Sheet Molding Compound
WF	Wood Fiber
WPC	Wood Plastic Composite
